# Plication of fascia transversalis in comparison to no plication to reduce seroma formation post laparoscopic transabdominal preperitoneal repair for direct inguinal hernia : a randomized controlled trial

**DOI:** 10.1007/s10029-026-03623-9

**Published:** 2026-03-17

**Authors:** Mohammed Elshwadfy Nageeb, George Abdelfady Nashed, Ali Abdelrahman Ali Asal, Khaled Mosleh Torfa

**Affiliations:** 1 Faculty of Medicine, Cairo University, Cairo, Egypt; 2 KasrAlainy Hospital, Cairo University, Cairo, Egypt

**Keywords:** Fascia transversalis, TAPP, Seroma, Directinguinal hernia, Plication

## Abstract

**Purposes:**

Seroma is a frequent complication after laparoscopic hernias repair. Many techniques have been proposed to mitigate this issue, yet no approach has been standardized. This study aimed to evaluate the effectiveness of transversalis fascia plication to reduce this.

**Methods:**

This randomized controlled trial was conducted at University Hospital from Jule 2024 to October 2025 after approval by the research ethics committee (MS-142-2024), involving adult patients who underwent TAPP repair for direct inguinal hernia. Exclusion criteria were indirect/pantaloon or recurrent hernias, conversion to open surgery, and concurrent procedures. Patients were randomized into two groups by ratio1:1 using computer-generated random sequence with opaque sealed envelopes opened only intraoperative after confirmation of direct hernia: Group A: underwent plication of the fascia transversalis, while the other did not. The primary outcome was the incidence of postoperative seroma. Secondary outcomes included postoperative recovery parameters and the complications. Secondary outcomes included postoperative recovery parameters and the complications.Statistical analysis was performed using SPSS version 27.

**Results:**

146 patients met the inclusion criteria. Demographic characteristics, preoperative comorbidities and hernia size were similar across both groups. At day 30 follow-up, 9 patients (12.3%) in the control group developed clinically detectable seromas, whereas none were in the plication group (p =0.002). Additionally, the plication group demonstrated a significantly faster return to normal activity and better pain scores.

**Conclusions:**

Plication of the transversalis fascia during TAPP may reduce the incidence of seroma formation, and improve postoperative outcomes. Before starting the study, the trial was registered and approved by institutional research ethics committee (MS-142-2024).online, retrospectively registered on the Pan African Clinical Trials Registry (PACTR202511671623081).

## Introduction

Laparoscopic transabdominal preperitoneal (TAPP) repair has become a widely accepted approach for inguinal hernia repair due to its advantages, including reduced post-operative pain, faster recovery compared to open techniques [1]. However, one of the most frequentl encountered complications following TAPP repair of direct inguinal hernias is the formation of post-operative seroma, which can cause patient discomfort, prolong recovery, and sometimes necessitate additional interventions [2].

Several approaches have been explored to minimize the risk of postoperative seroma, including the use of drains, compression dressings, and technical modifications. To date, no standardized method has been universally adopted. Among the proposed strategies, plication of the transversalis fascia has gained attention as a means of obliterating dead space (so decrease the space for seroma formation), reinforcing **the posterior wall**, which potentially decrease recurrence rates, this was observed in **robotic posterior wall reinforcement** techniques. demonstrated that reinforcement of posterior wall—was associated with less seroma and recurrence. transversalis fascia plication offers a cost-effective and widely applicable step to achieve these benefits. While some reports indicate that defect closure may lower seroma rates and improve long-term pain control, others have expressed concern about possible increases in operative time or recurrence risk [3, 4].

The aim of this study was to evaluate the role of transversalis fascia plication in reducing seroma formation following laparoscopic transabdominal preperitoneal (TAPP) repair of direct inguinal hernia.

## Materials and methods

### Study design

This study was designed as a single center prospective double-parallel, randomized clinical trial. It was conducted at general surgery department, Kasr Al-Ainy Hospital, Cairo University, from jule 2024 to October 2025.

The InstitutionalResearch Ethics Committee (REC) was approved before starting the study (identifier: MS142-2024). The study was retrospectively registered at the Pan African Clinical Trail Registry Identifier: PACTR202511671623081.The study followed the CONSORT guidelines. All patients provided written informed consent and had the right to withdraw anytime during the study.

Sample Size and Power.

Sample size calculated depending on a previous study by Ng et al. **[**5] as reference. According to this study, when presence of seroma in one group was 17.5% while in the other group it was 2.1%, when the (power) is 0.8 and the Type I error probability associated with this test 0.05.The minimum sample size needed is 55 cases per group. Total sample size increased to 66 to compensate for 20% drop out percentage. The sample size was calculated using Chi Square test which was performed by using P.S.Power 3.1.2., the calculated minimum sample size was 66 patients in each group. Ultimately, 73 patients were recruited in each group to further enhance the robustness and statistical reliability of the findings and to account for any potential unforeseen data loss or protocol deviations.

The study included adult patients (> 18 years) diagnosed clinically with direct inguinal hernia (clinical diagnosis by internal ring test, Patients presenting with a groin swelling with an impulse on cough that reprotruted after reduction with closure of internal ring by finger) who were scheduled for TAPP mesh hernioplasty.

The following patients were excluded from the study:

- Patients with indirect or pantaloon inguinal hernia (discovered.

Intraoperatively, after peritoneal incision and identification of the sac’s position relative to the inferior epigastric vessels — medial (direct) or lateral (indirect). )

- Patients with recurrent hernia.
- Conversion to open surgery.
- Undergoing concurrent surgical procedures.
- only cases with **bilateral direct hernias** were included in the final analysis. Patients found intraoperatively to have any indirect component on either side were excluded.

### Randomization

Randomization process was adherent to CONSORT guidelines.

- **Sequence generation**: A computer-based random sequence was created using Randomizer.org, assigning patients in a 1:1 ratio to either the plication or non-plication group.
- **Allocation concealment**: Sequentially numbered, sealed, opaque envelopes were prepared by an independent investigator assistant who was not involved in patient recruitment, the operation, or outcome assessment.
- **Implementation**: intraoperatively, after confirming the diagnosis of a direct hernia The envelope was opened to enroll the patient to one group.

#### Blinding

The operating surgeon was aware of group allocation (as plication is a procedural step), but both the **patients** and the **postoperative outcome assessors** were blinded to group assignment. The assessors—independent surgeons who were not part of the operative team—performed all postoperative clinical evaluations and data analysis without access to allocation records.

The patients, the outcome assessors and investigators were blinded.

The patients were divided into two groups:

- Group A: patients underwent standard TAPP repair with fascia transversalis plication.
- Group B: patients underwent standard TAPP repair without plication.

### Preoperative care

All candidates were subjected to thorough history taking, clinical examination and withdrawn the indicated laboratory investigations.

The diagnosis of direct inguinal hernia was clinically with (clinical diagnosis by internal ring test, Patients presenting with a groin swelling with an impulse on cough that reprotruted after reduction with closure of internal ring by finger). However, **f**inal confirmation of hernia type was made intraoperatively after peritoneal incision and identification of the sac’s position relative to the inferior epigastric vessels — medial (direct) or lateral (indirect).

**Hernia size was assessed clinically** according to the traditional anatomical extent of the hernia sac:

- **Bubonocele**: the hernia sac is limited to the inguinal canal and does not reach the external ring;
- **Funicular**: the hernia sac extends beyond the superficial ring but does not descend into the scrotum;
- **Inguinoscrotal**: the hernia sac extends into the scrotum.

### Surgical procedure

All patients underwent laparoscopic transabdominal preperitoneal (TAPP)hernia repair under general anesthesia using a 15° Trendelenburg position.

• A standard 3-port technique was employed, with ports positioned at the.

Umbilical level.

• After creating a peritoneal incision, the preperitoneal space was carefully.

dissected and the hernia sac was reduced.

**Objective intraoperative hernia defect measurements**, recorded using laparoscopic calipers after peritoneal incision.

**Fig. 1:**
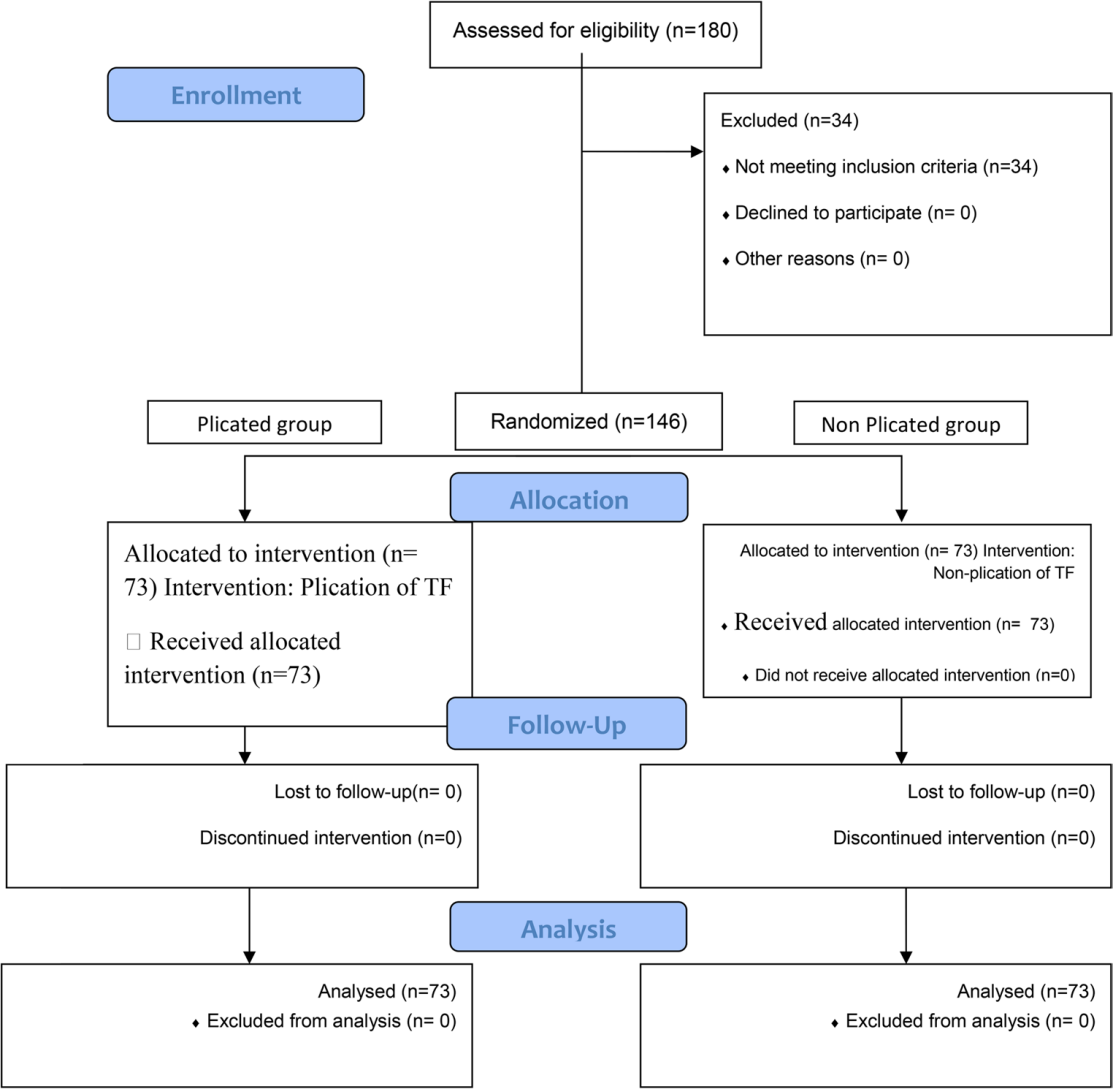
Flowchart of the study

In Group A (plication group), (Fig. 1) after the hernia sac was completely, the **evaginated transversalis fascia was plicated using a continuous 2 − 0 polyfilament non-absorbable suture **applied in **1–3 rows depending on defect width**. The plication was directed **from superior edge of the defect to the inferior edge**, thereby restoring the posterior wall contour and eliminating the potential dead space and ensuring tension-free apposition and uniform reinforcement (Fig. 1). The step was facilitated by gentle external groin pressure applied by the assistant. Following successful plication, a light weight polypropylene mesh was positioned to cover the myopectineal orifice and secured with three absorbable sutures (Polyglactin 3 − 0): two medially and one laterally. The peritoneum was then closed using a continuous running suture.

In Group B, the TAPP procedure was performed in the same manner, except that plication of the transversalis fascia was omitted. Mesh placement and fixation were carried out identically.

### Postoperative care

Patients were monitored overnight for vital stability and received standard analgesia (paracetamol 1 g every 8 h). Oral intake was initiated four hours after surgery.

### Discharge criteria

1. Hemodynamically stable.
2. Able to tolerate oral intake.
3. Ambulating independently.

Patients were prescribed the same analgesia (Paracetamol 1 gm/PRN).

### Follow-up

the postoperative assessments were scheduled at day 1 (prior to discharge), day 10 (for suture removal), and day 30 (final evaluation). Patients were also instructed to return if they experienced delayed complications such as seroma, persistent pain, recurrence, or swelling.

### Outcome measures

The primary endpoint was the incidence of postoperative seroma.The presence of seroma was diagnosed clinically (by detection of a groin swelling or mass that has no impulse on cough) and confirmed by ultrasonography (Sac containing collection with no vascularity).

The secondary outcomes included, Pain (VAS score at 1, 10 and 30 days), incidence of complications (e.g., bleeding, infection, urinary retention), hernia recurrence within 30 days.

### Data collection and statistical analysis

For all participants, demographic and clinical data were collected, including:

- Age, gender, and BMI.
- Comorbidities and current medications.
- Hernia side and defect size.
- Type of mesh used.
- Duration of surgery.
- Intraoperative and postoperative complications.

### Statistical data analysis

Data were analyzed using IBM SPSS version 25. Descriptive statistics.

(mean} SD for continuous variables; frequencies and percentages for.

categorical variables) were reported. Comparisons between the two groups wereconducted using the unpaired t-test for continuous variables and the Chi-square.

test for categorical variables. A p-value < 0.05 was considered statistically.

significant.

## Results

**Fig. 2:**
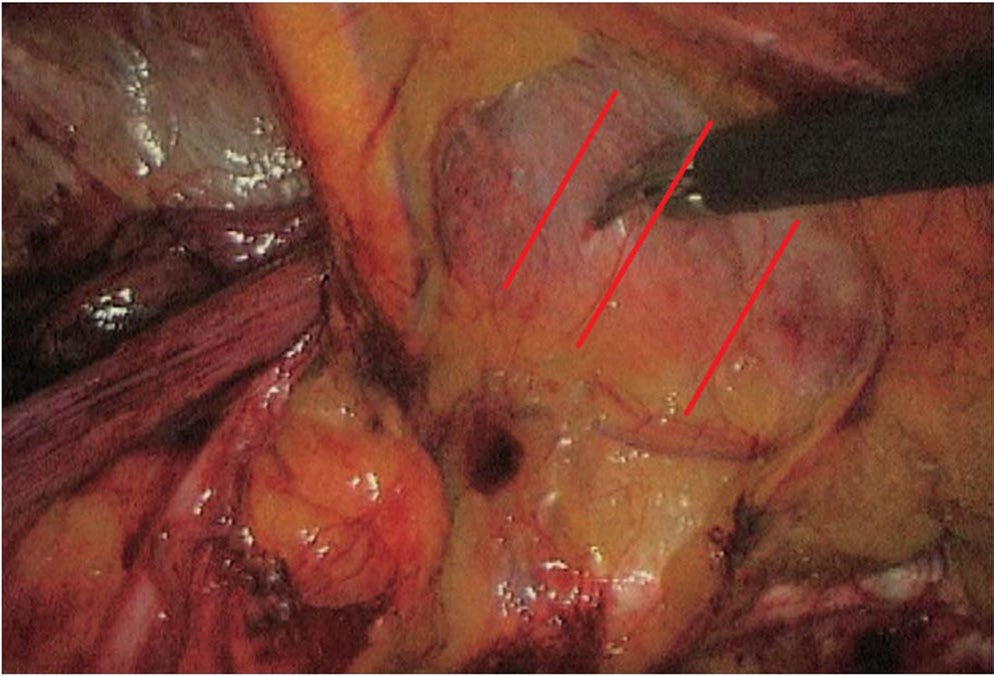
The red line showing the direction of the plication rows

The study included 180 patients assessed for eligibility with 146 of them meeting the inclusion criteria. 34 patients were excluded due to: 17 patients were discovered as indirect hernia intraoperative, 15 were pantaloon hernia and 2 were converted to open. 73 patients underwent plication of fascia transversalis and 73 patients were included in the *non-plicated group*. All Patients were followed up. No patients were lost during the follow-up (Fig. 2).

I. Demographic data:

All the study patients were males. The mean age of the *Plication* group was 50.60 ± 8.28 years, while that of the *No Plication* group was 49.08 ± 12.49 years, with a mean difference of 1.52 ± 1.75 years (95% CI: −1.95 to 4.99, *p* = 0.39).Similarly, the mean BMI was 27.63 ± 2.89 kg/m² in the *Plication* group and 27.05 ± 2.94 kg/m² in the *No Plication* group, with a mean difference of 0.58 ± 0.48 (95% CI: −0.37 to 1.54, *p* = 0.23) (Table 1).

**Table 1:** Demographic data of both groups:

	Group				Independent t test				
	Plication		No Plication		Mean Difference	Std. Error Difference	95% Confidence Interval of the Difference		*P* value
	Mean	Standard Deviation	Mean	Standard Deviation			Lower	Upper	
Age	50.60	8.28	49.08	12.49	1.52	1.75	-1.95	4.99	0.39
BMI	27.63	2.89	27.05	2.94	0.58	0.48	-0.37	1.54	0.23

II. Preoperative data:

There were no statistically significant differences between the Plication and No Plication groups regarding preoperative albumin (4.18 ± 0.18 vs. 4.19 ± 0.19 g/dL, *p* = 0.688) or hernia defect size (1.55 ± 0.76 vs. 1.75 ± 0.78 cm, *p* = 0.136).Similarly, the two groups showed no significant differences in smoking status (*p* = 0.55), anticoagulant use (*p* = 0.56), previous groin surgery (*p* = 1.00), bilaterality (*p* = 1.00), hernia side (*p* = 0.88), hernia size type (*p* = 0.96), diabetes mellitus (*p* = 0.90), or hypertension (*p* = 0.91) (Tables 2)

**Table 2:** Preoperative data of both groups

		Group				*P* value
		Plication		No Plication		
Preoperative Albumin (g/dL) M ± SD		4.18	0.18	4.19	0.19	0.688
Defect Size (cm) M ± SD		1.55	0.76	1.75	0.78	0.136
Smoking Status N(%)	no	58	79.5%	55	75.3%	0.55
	yes	15	20.5%	18	24.7%	
Anticoagulant Use N(%)	no	71	97.3%	72	98.6%	0.56
	yes	2	2.7%	1	1.4%	
Previous Groin Surgery N(%)	no	73	100.0%	73	100.0%	
Bilateral Hernia N(%)	no	63	86.3%	63	86.3%	1
	yes	10	13.7%	10	13.7%	
Hernia side N(%)	right	33	45.2%	35	47.9%	0.88
	left	28	38.4%	28	38.4%	
	bilateral	12	16.4%	10	13.7%	
Hernia type N(%)	direct	73	100.0%	73	100.0%	----
Hernia Size Type N(%)	Funicular	32	43.8%	32	43.8%	0.96
	bubonocele	34	46.6%	33	45.2%	
	inguinoscrotal	7	9.6%	8	11.0%	
Diabetes Mellitus N(%)	No	58	79.5%	49	80.3%	0.9
	Yes	15	20.5%	12	19.7%	
Hypertension N(%)	No	58	79.5%	48	78.7%	0.91
	Yes	15	20.5%	13	21.3%

III. Operation data:

73 patients underwent plication of fascia transversalis and 73 patients were included in the *non-plicated group* Both groups used lightweight mesh with suture fixation exclusively.

The mean operative duration was 109.13 ± 27.77 min in the *Plication* group and 108.36 ± 24.57 min in the *No Plication* group, with no significant difference between them (*p* = 0.80).

IV. Postoperative outcomes:

the incidence of seroma was significantly higher in the No Plication group compared with the Plication group at all time points, Day 2 (19.2% vs. 2.7%, *p* = 0.001), Day 10 (16.4% vs. 2.7%, *p* = 0.005), and Day 30 (12.3% vs. 0.0%, *p* = 0.002) (Table 3).

**Table 3:** Postoperative outcomes of both groups

Post operative		Group *N*(%)				*P* value
		Plication		No Plication		
Hospital Stay (days)	1 day	73	100.0%	73	100.0%	----
Seroma at Post-op Day 2	No	71	97.3%	59	80.8%	0.001*
	Yes	2	2.7%	14	19.2%	
Seroma at Day 10	No	71	97.3%	61	83.6%	0.005*
	Yes	2	2.7%	12	16.4%	
Seroma at Day 30	No	73	100.0%	64	87.7%	0.002*
	Yes	0	0.0%	9	12.3%	
Chronic Pain at Month 3 (1/0)	No	73	100.0%	73	100.0%	----
Hernia Recurrence at 30 Days (1/0)	No	73	100.0%	72	98.6%	0.32
	Yes	0	0.0%	1	1.4%	
Post-op Complications	No	73	100.0%	72	100.0%	-----

The subgroup of patients with large direct hernia defects (> 2 cm), only 8 patients had defect size more than 2 cm, four patients in each group. A transient early seroma occurred in one patient 1/4 (25%) from the non-plication group on postoperative day 2, which resolved spontaneously by day 10, with no residual or late seromas at 30 days. In contrast, no seromas were recorded in any of the four patients within the plication group throughout the follow-up period (Days 2, 10, and 30) (Table 4).

**Table 4:** Subgroup analysis – hernia with defects > 2 cm

Time Point	Plication		Non-Plication		Plication	Non-Plication
	Seroma (Yes)	Seroma (No)	Seroma (Yes)	Seroma (No)	Seroma Rate (%)	Seroma Rate (%)
Day 2	0	4	1	3	0	25
Day 10	0	4	0	4	0	0
Day 30	0	4	0	4	0	0

Both groups had identical hospital stays of 1 day postoperatively.

**Fig. 3:**
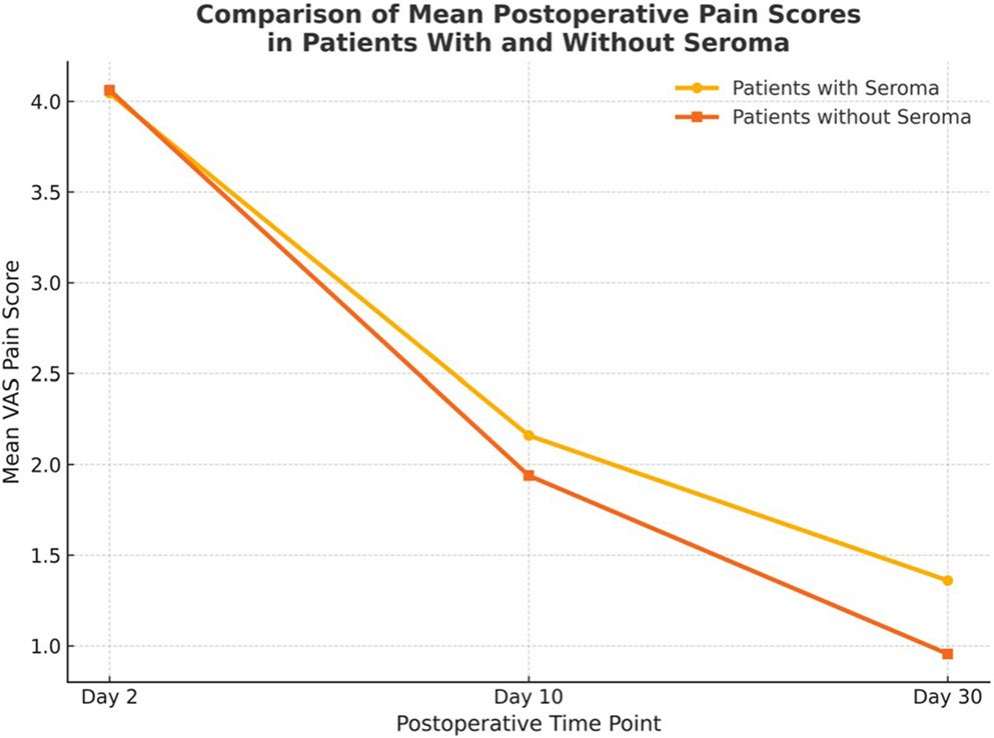
Correlation between seroma formation and postoperative pain

Pain scores were significantly lower in the Plication group compared with the No Plication group at all postoperative time points (day 2, 10 and 30).These results indicate a consistently lower postoperative pain level in the Plication group throughout the follow-up period (Table 5). However, There were no cases of chronic pain in either group at 3 months. Correlation analysis between postoperative pain and seroma formation revealed that patients who developed seromas demonstrated notably higher VAS pain scores at postoperative days 10 and 30 compared with the mean values of their respective groups. These findings are illustrated in Fig. 3, which shows mean VAS pain scores with standard deviations at days 2, 10, and 30 for both groups, highlighting the overlap between seroma presence and elevated pain in the non-plication group ( Fig. 3).

**Table 5:** Pain score of both groups

Pain Score	Group M ± SD				Mean Difference	Std. Error Difference	95% Confidence Interval of the Difference		*P* value
	Plication		No Plication				Lower	Upper	
Post-op Day 2	4.080	0.000	4.040	0.000	0.04	0.0	0.04	0.04	< 0.0001*
At Day 10	1.680	0.000	2.240	0.000	-0.56	0.0	-0.56	-0.56	< 0.0001*
At Day 30	0.600	0.000	1.360	0.000	-0.76	0.0	-0.76	-0.76	< 0.0001*

Hernia recurrence occurred in only one patient (1.4%) in the No Plication group, with no significant difference between groups (*p* = 0.32).

No other postoperative complications were reported.

## Discussion

Laparoscopic repair of the inguinal hernia is widely used due to its advantages over the open techniques. Nevertheless, postoperative seroma remains a recognized complication following TAPP repair of direct inguinal hernias, which can cause patient discomfort, prolong recovery, and sometimes necessitate additional interventions [2].

Current strategies to reduce the occurrence of seroma can be broadly divided into two main groups: by placing a closed-system suction drain after surgery or by eliminating the dead space using different techniques [6].Controversies still exist regarding which method is the best. Plication of fascia transversalis has emerging to be one of the tools to reduce seroma formation [7].

This prospective study included 146 patients with direct inguinal hernia undergoing laparoscopic TAPP repair at university tertiary care Hospital, randomized into two equal groups: those who underwent plication (*n* = 73) and those who did not (*n* = 73). All participants were male. These results indicate no statistically significant differences between the two groups regarding age or BMI, confirming that both groups were demographically comparable at baseline.

Baseline distribution of right- and left-sided hernias and mean hernia defect size were well balanced (1.55 ± 0.76 vs. 1.75 ± 0.78 cm, *p* = 0.136) .There were no statistically significant differences between both groups regarding smoking status, anticoagulant use, previous groin surgery, bilaterality, hernia size type, diabetes mellitus, hypertension or preoperative albumin .These findings indicate that both groups were clinically comparable at baseline, minimizing confounding factors.

Regarding operative parameters, both groups showed comparable operative times (**109.13 ± 27.77 min** in the *Plication* group and **108.36 ± 24.57 min** in the *No Plication* group, *p* = 0.80),suggesting that the addition of plication did not significantly extend the duration of surgery. All patients in both groups had mesh fixation with sutures, maintaining procedural consistency except for the plication intervention.

In our study, the post-operative outcomes revealed several significant advantages in the plicated group. A significant outcome in our study was the faster return to normal activity in the plicated group (6.52 ± 1.23 vs. 8.08 ± 2.06 days, *p* = 0.007). This is consistent with findings from Furtado et al., (2019) who suggested that securing the pseudo sac during TAPP repair can improve postoperativerecovery by enhancing anatomical integrity [8]. Our results reinforce the idea that transversalis fascia plication contributes to better functional recovery, potentially due to improved structural support and reduced post-operative fluid accumulation.

One of the key findings of our study was the significant reduction in seroma formation in the plicated group, compared to the non-plicated group. the incidence of seroma was significantly higher in the No Plication group compared with the Plication group at all time points, Day 2 (19.2% vs. 2.7%, *p* = 0.001), Day 10 (16.4% vs. 2.7%, *p* = 0.005), and Day 30 (12.3% vs. 0.0%, *p* = 0.002). at postoperative day 30, there was no cases of seroma in the plicated group but there were 9 cases in non plicated group(12.3%). The subgroup of patients with large direct hernia defects (> 2 cm), only 8 patients had defect size more than 2 cm, four patients in each group. A transient early seroma occurred in one patient 1/4 (25%) from the non-plication group on postoperative day 2, which resolved spontaneously by day 10, with no residual or late seromas at 30 days.

In contrast, no seromas were recorded in any of the four patients within the plication group throughout the follow-up period (Days 2, 10, and 30).

Although this subgroup size is small and not powered for formal statistical comparison, the trend observed reinforces the protective effect of fascia transversalis plication, particularly in larger direct defects where residual dead space is more substantial. The reduction in seroma formation supports the hypothesis that plication eliminates dead space, reducing the likelihood of fluid accumulation.

Additionally, there was one case of early recurrence (within 3 weeks) in the non-plicated group compared to none in the plicated group, although this difference was not statistically significant (*p* = 0.32) but this support supports the mechanical rationale that plication effectively obliterates potential spaces and reinforces the posterior wall. This aligns with the findings of Pini et al., (2021) who reported a significant reduction in seroma formation when the transversalis fascia was anchored to the Cooper ligament during robotic-assisted TAPP repair, suggesting that additional reinforcement techniques may enhance post-operative outcomes [4].

Similarly, Singh and Rajyaguru (2023) demonstrated that a pre-tied suture loop technique for direct hernia defect closure was effective in preventing seromas without increasing operative time or recurrence rates [9].

This complements the results of Lodha et al. (2023) who introduced a newer technique of reducing seroma formation by fenestration of the pseudo-sac (thickened transversalis fascia) in patients undergoing laparoscopic hernia repairfor uncomplicated direct inguinal hernia [10].

This is consistent with the findings of Furtado et al., (2019) who noted that large direct defects may benefit from additional fixation strategies to minimize the risk of recurrence [11].

In our study, Pain outcomes further highlight the potential benefit of plication. Although early postoperative pain was similar between groups, patients in the plicated group reported significantly lower pain scores at 30 days (0.60 ± 0.50 vs. 1.36 ± 0.57, *p* < 0.0001). The positive correlation between seroma formation and higher postoperative pain indicates that localized inflammatory tension contributes to early discomfort.This supports the notion that plication may reduce chronic post-herniorrhaphy pain, likely through improved anatomical restoration and decreased inflammatory response. Abraham (2020) similarly observed that disruption of the transversalis fascia contributes to long-term discomfort, and that reinforcement techniques enhance durable pain control.

### Limitations of the study

Although our study showed a significant reduction in seroma formation, it is important to emphasize that seroma is primarily an early postoperative complication, which was the primary endpoint of the study. For this reason, we selected a 30-day follow-up period, as it is generally sufficient to capture all cases of postoperative seroma after laparoscopic TAPP repair.

At the same time, we acknowledge that our study’s single-center nature, modest sample size, and short follow-up period for outcomes such as recurrence and chronic pain may limit how broadly these findings can be applied. To strengthen future research, we suggest that larger, multicenter trials with longer follow-up be conducted to confirm these results and to explore the long-term effectiveness, chronic pain, recurrence rates, and cost implications of fascia transversalis plication in different surgical environment.

## Conclusion

Our findings suggest that transversalis fascia plication during TAPP repair of direct inguinal hernias is associated with a reduction in seroma formation at the same operative time, faster recovery, and reduced long-term pain. Further large-scale, randomized studies are needed to confirm these findings and determine the optimal approach for direct inguinal hernia repair.

## Data Availability

All datasets are collected on a spreadsheet and available from the corresponding author upon reasonable request.
